# In-Operando Diffraction and Spectroscopic Evaluation of Pure, Zr-, and Ce-Doped Vanadium Dioxide Thermochromic Films Derived via Glycolate Synthesis

**DOI:** 10.3390/nano10122537

**Published:** 2020-12-17

**Authors:** Stanislav Kurajica, Vilko Mandić, Ivana Panžić, Mattia Gaboardi, Katarina Mužina, Ana Lozančić, Juraj Šipušić, Ivana Katarina Munda, Lucija Višić, Sanja Lučić Blagojević, Lara Gigli, Jasper Rikkert Plaisier

**Affiliations:** 1 Faculty of Chemical Engineering and Technology, University of Zagreb, Marulićev trg 20, 10000 Zagreb, Croatia; stankok@fkit.hr (S.K.); kmuzina@fkit.hr (K.M.); alozanc@irb.hr (A.L.); jsipusic@fkit.hr (J.Š.); imunda@fkit.hr (I.K.M.); lvisic@fkit.hr (L.V.); slucic@fkit.hr (S.L.B.); 2 Ruđer Bošković Institute, Bijenička Cesta 54, 10000 Zagreb, Croatia; ipanzic@irb.hr; 3 Elettra Sincrotrone Trieste S.C.p.A., Strada Statale 14, 4149 Trieste, Italy; mattia.gaboardi@elettra.eu (M.G.); lara.gigli@elettra.eu (L.G.); jasper.plaisier@elettra.eu (J.R.P.)

**Keywords:** smart window, vanadium dioxide, Zr-doping, Ce-doping, thermochromic transition, structural properties, IR transmission/reflection, in-operando, GIXRD-Raman f(T) setup, multi-channel characterisation

## Abstract

Pure and doped vanadia (VO_2_, V_0.98_Zr_0.02_O_2_, V_0.98_Ce_0.02_O_2_) samples were prepared by wet chemistry synthesis from vanadyl glycolate intermediate phase and tape casted into films. Combining in-operando grazing incidence synchrotron X-ray diffraction and Raman spectroscopy, we studied the structural evolution of the films under isothermal conditions. The setup allowed assessment of the thermochromic functionality with continuous monitoring of the monoclinic to tetragonal transition in pure and doped vanadia phases, responsible for the transmission and reflection of light in the infrared part of the solar spectrum. The materials characterisation by X-ray diffraction beamline (MCX) goniometer demonstrated ideal performance, combining flexible geometry, high resolution, and the potential to accommodate the multi-channel equipment for in-operando characterisation. This method proved viable for evaluating the relevant structural and physical, and thereof functional properties of these systems. We revealed that dopants reduce the transition temperature by 5 °C on average. The synthetic route of the films was held responsible for the observed phase separation. The more favourable behaviour of cerium-doped sample was attributed to cerium alkoxide behaviour. In addition, structural, microstructural, thermal, and spectroscopic characterisation on powder samples was performed to gain more insight into the development of the phases that are responsible for thermochromic features in a broader range of doping ratios. The influence of the dopants on the extent of the thermochromic transition (transmission to reflection hysteresis) was also evaluated using (micro) structural, thermal and spectroscopic methods of powder samples. Characterisations showed that zirconium doping in 2, 4, and 6 mol% significantly influenced the phase composition and morphology of the precursor. Vanadium oxides other than VO_2_ can easily crystallise; however, a thermal treatment regime that allowed crystallisation of VO_2_ as a single phase was established.

## 1. Introduction

Vanadium dioxide (VO_2_) is a thermochromic material that undergoes a reversible phase transition at 68 °C from a low-temperature monoclinic and semiconductor phase, that is permeable to infrared radiation, into a high-temperature tetragonal-rutile metallic phase, that is semi-permeable to infrared radiation [1]. Thanks to this transformation, VO_2_ can be used as a key element of smart windows simply by depositing thermochromic films onto the glass. Under specific conditions, a thermochromic window will reflect part of the infrared (IR) radiation, while transmitting the visible (VIS) one.

These thermochromic smart-windows can be used to achieve solar radiation selectivity, e.g., where the energy efficiency of a building can be increased owing to the reduction in energy consumption for cooling systems [2]. While VO_2_ is considered to have many advantages over other inorganic and organic thermochromic materials, there are practical issues that hinder its widespread application. The main shortcomings of VO_2_ are the following: (1) high critical temperature, *T_c_*, of the phase transformation (68 °C) that prevents building application; (2) low luminous transmittance, *T_lum_*, (ratio of transmitted and incident visible radiation), which limits the VO_2_ layer thickness; (3) limited solar modulation ability, Δ*T_sol_*, (difference in permeability of total solar radiation of metal and semiconductor phases); and (4) unpleasant yellowish colour [3]. The strategy to modify VO_2_ by doping with different metals such as W, Mo, Ti, Zr, etc., may be feasible for reducing *T_c_* while increasing *T_lum_* and Δ*T_sol_* and improving the visual appearance [4,5].

Although none of the single dopants alone can solve all of the aforementioned drawbacks, doping with Zr is considered to be acceptable solution. The difference in the radii between V^3+^ and Zr^4+^ is sufficient to: (i) destabilize the monoclinic VO_2_ structure, leading to a decrease in *T_c_*, (ii) expand the bandgap of VO_2_, which can lead to a rise in *T_lum_* and Δ*T_sol_*, and (iii) affect the colour [6]. As an example of successful doping, Lu et al. [7] showed that Zr doping with up to 4% of VO_2_ films lead to a lower phase transition temperature at about 50 °C. Literature data on Zr doping of VO_2_ is widely available [8]. Work by Zong at al. showed that the luminous transmittance and solar modulation ability of doped VO_2_ films can be improved by employing more complex configurations, such as those with SnO_2_ buffer layers [9]. Here, we focus on simple monolayer doped VO_2_ films, rather than on such complex configurations.

Another interesting dopant element that may reduce all the technological obstacles is Cerium. Cases of Ce doping have been reported for different systems; however, Ce is only marginally considered for VO_2_ films, often in complex configurations [10,11,12]. Song et al. reported one of the few considerations for Ce-doped VO_2_ monolayer films with promising thermochromic applicability [13].

Doped VO_2_ is commonly prepared by hydrothermal synthesis [6], chemical vapour deposition [14], magnetron sputtering [15], sol–gel synthesis [16], etc. Although these methods usually require long-lasting thermal treatment at high temperatures in an inert atmosphere, VO_2_ films can be successfully prepared by fairly low temperature magnetron sputtering depositions [17,18,19,20]. Nevertheless, VO_2_ can be prepared by means of facile wet-chemistry synthesis through intermediate vanadyl glycolate, VO(OCH_2_CH_2_O), thus enabling short annealing times and avoiding inert atmosphere requirements [21].

In this manuscript, we investigated, in operando, three different deposited films (VO_2_, Zr-VO_2_ and Ce-VO_2_) by carrying out, in situ, Grazing Incident synchrotron X-ray Diffraction (GIXRD), synchrotron X-ray Reflectivity (XRR), and Raman spectroscopy at various temperatures. Capitalising on the outcome of this model experiment, we acknowledge our intention to show the feasibility of the advanced characterisation setup using the highly versatile MCX diffraction beamline for probing at the nanoscale the functional properties of specific systems such as thermochromic windows. Furthermore, in this article, we report on the synthesis of the same compositions in form of powders, followed by a thorough characterisation by means of powder X-ray diffraction (XRPD), thermal analyses (DTA/TGA/DTGA/DSC), Scanning Electron Microscopy (SEM), and vibration spectroscopy (FTIR). This complementary section is meant to provide better insight into the preparation of thermochromic materials and the assessment of their properties.

## 2. Materials and Methods

### 2.1. Synthesis

#### 2.1.1. Powders

The synthesis procedure for preparing pure vanadia has previously been published [21]. Zr-doped and Ce-doped vanadium dioxide syntheses followed the same principle. Briefly, 0.025:1:0.005 of ammonium metavanadate, NH_4_VO_3_ (p.a. Merck, Darmstadt, Germany), ethylene glycol C_2_H_6_O_2_ (p.a. Kemika, Zagreb, Croatia), and dopant were mixed together and refluxed at 160 °C. Dopant precursors were zirconium butoxide, Zr(OC_4_H_9_)_4_ (p.a. Merck, Darmstadt, Germany), and cerium acetylacetonate hydrate, Ce(O_2_C_5_H_7_)_3_·H_2_O (p.a. Alfa Aesar, Haverhill, MA, USA). The precipitates were processed by multistep centrifugation and C_2_H_6_O_2_ rinsing to yield intermediate vanadyl glycolate, VO(OCH_2_CH_2_O) slurries, which were dried in an ambient atmosphere and box convection dryer at 150 °C for 2 h. Samples were stored for subsequent thermal treatment (see Table 1).

#### 2.1.2. Films

Slurries were pre-dried in a vacuum furnace at 100 °C for 1 h. To obtain films with consistent selected thicknesses using tape casting with selected blade profiles, it was necessary to optimise the thermochromic to binder material ratio. Then, precursor materials were non-stoichiometrically diluted using 12 wt.% solution of PVDF (Poly(1,1-difluoroethylene), [CH_2_CF_2_]*_n_*, p.a. Sigma Aldrich, St. Louis, MO, USA) in NMP (1-Methylpyrrolidin-2-one, C_5_H_9_NO, p.a. Sigma Aldrich, St. Louis, MO, USA) and homogenised in a mortar until empirically viscous enough for the tape casting. Tape casting was performed using doctor’s blade (Qualtech Products Industry, Denver, CO, USA) with blade gaps from 10, 20, 40, and 80 μm. The selected film thickness was not optimized in terms of film functionality; rather, it was pragmatically selected to remain in the thin-film configuration while enabling sufficient thickness to facilitate characterisation. In addition to thickness selection, preparing films by tape casting allows facile preparing of films over large surfaces, which is interesting for process scale-up. Conventional microscopy glass slides were used as substrates. Prepared films were dried in vacuum furnace at 100 °C for 6 h (Table 1).

### 2.2. In-Operando Film Characterisation

In situ performance was investigated using synchrotron radiation at the MCX beamline at the Elettra Synchrotron facility (Trieste, Italy) [22] with a self-developed multifunctional in situ cell for simultaneous measurement of GIXRD, XRR, and Raman spectroscopy as a function of temperature on multi-layered thin films. In the centre of the four-axis Huber goniometer, a resistor-heated (three serial CQC6R8J 9W resistors), air convection-cooled aluminium hotplate (hollow Al 8 × 6 × 2 cm box, resistors glued by silicone thermo-paste inside at the ceiling position for heating and glass wool at bottom for insulation) was mounted on the MCX sample stage, further separated by an insulating 5-mm-thick Teflon disc. Heating cell temperature was regulated by a HWE 12 V DC thermistor-based controller. A multichannel Siglent SPD3303S power supply device ensured a 12 V DC voltage to the temperature controller and a fixed 14 V DC voltage (21 W) to the resistor series. The film temperature was measured by a K-type thermocouple via the multichannel Pico TC-08 data logger. Planar samples were positioned flat on the hotplate surface. The hotplate stage was centred on the goniometer exploiting the direct beam using *z*-scan and *θ*-scan routines. Measurements were performed isothermally in air; relative humidity was maintained at 20%. For RT measurements, the temperature was maintained at 25 °C. The Raman optical sensor was mounted vertically to the hotplate stage and focused at 7 mm distance. The probe was sustained on the sample stage so that the beam remained at focus during the whole experiment. Samples were excited using a PD-LD LS-2 100 mW laser at a wavelength of 635 nm. Scans were collected using the Maya2000Pro Ocean Optics device in the range 100–1200 cm^−1^ with 10 s collecting time. GIXRD was performed using a monochromatic 8 keV beam (spot size approximately 300 × 200 μm) at several grazing angles (0.40, 0.75, 1.50) in the 2*θ* range 15–45°, with continuous steps 0.01° 2*θ* and collecting time of 0.2 s (receiving slits 300/400 µm). XRR was recorded in the range of 0–7° 2*θ*, with steps 0.01° 2*θ* and a collecting time of 0.2 s. Isothermal measurements were performed at 60–100 °C with Δ*T* = 5 °C. Rietveld refinement of data was carried out using GSAS-2 suite (Chicago, IL USA) [23]. A silicon standard was exploited to fit the instrumental profile. Patterns were refined sequentially over the available temperature range using the phases discussed in Section 3. The profile shape was simulated accounting for the standard model for isotropic domain-size broadening, while microstrain effects were considered negligible. Only phase fractions and lattice parameters were refined, whereas thermal parameters and occupancies were kept at a fixed value to avoid unwanted correlations.

### 2.3. Powder Characterisation

The X-ray powder diffraction (XRPD) was performed using a Shimadzu diffractometer XRD 6000 (Kyoto, Japan) with Cu-*Kα* radiation. Data were collected in a step scan mode with steps of 0.02° 2*θ* and counting time of 0.6 s.

IR spectroscopy (FTIR) was performed using a Bruker Vertex 70 (Billerica, MA, USA) in ATR (attenuated total reflectance) mode. Samples were pressed on a diamond and the absorbance data were collected between 400 and 4000 cm^−1^ with spectral resolution of 1 cm^−1^ and 64 scans.

Raman spectroscopy measurements were performed using a HORIBA Jobin Yvon T64000 spectrometer (Kyoto, Japan) with a 532.5 nm solid-state laser excitation. The spectra were collected in micro- Raman mode with a multi-channel CCD detector, laser power of 20 mW at the sample, and an objective with a 50× magnification (Olympus) in the range 0–1200 cm^−1^.

The morphologies were investigated using a Tescan Vega 3 scanning electron microscope (SEM, Brno, Czech Republic) operating at 30 kV. Samples for SEM characterisation were fixed on a sample holder using double-sided carbon conductive tape and then coated with gold using the Quorum SC 7620 sputter coater (Lewes, UK).

Thermal properties of the as-prepared samples were analysed using the simultaneous differential thermal analysis and thermos-gravimetric analysis (DTA/TGA) apparatus Netzsch STA 409C (Selb, Germany) at a heating rate of 10 °C min^−1^ in synthetic airflow of 30 cm^3^ min^−1^ with α-alumina used as a reference. Differential scanning calorimetry (DSC) apparatus Mettler Toledo DSC 823e (Columbus, OH, USA) was also used and calibrated with indium.

## 3. Results and Discussion

### 3.1. In-Operando Characterisation of Films

#### 3.1.1. Setup

**Figure 1:**
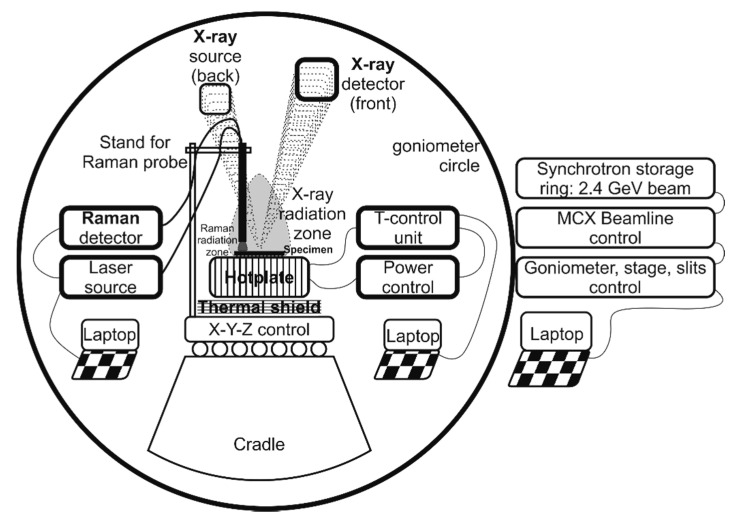
Scheme of the in-operando setup used on the MCX goniometer.

In-operando GIXRD was successfully used for revealing the specificities of the investigated vanadia thermochromic system. The experimental setup on the MCX beamline is shown in Figure 1. GIXRD and XRR measurements can be performed at precise spots on the specimens, in a wide range of 2*θ* between 0–80° at different *θ* angles, as a function of temperature. This particular setup enables the study of qualitative and semi quantitative crystalline phase composition, structural changes triggered by temperature, depth profiling of planar specimens, film thickness, morphological features such as crystallite size, preferred orientation, stress and strain in films, etc.

#### 3.1.2. What Can We See at Room Temperature?

**Figure 2:**
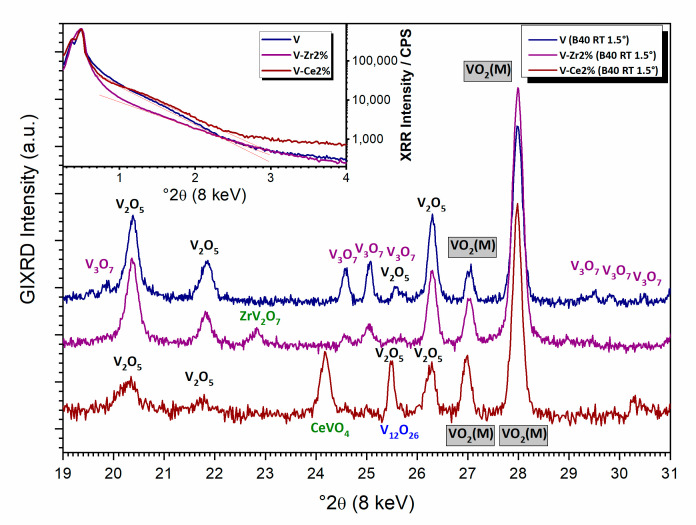
In-operando GIXRD results at 1.5° *θ* of all samples at room temperature. Inset: XRR scans for all samples.

With the GIXRD geometry, it is possible to obtain average information of the film’s crystallinity. At RT, the data collected from three specimens at 1.5° theta show predominately the presence of monoclinic vanadia phase VO_2_(M), assigned to ICDD PDF#43-1051, plus a minor fraction of orthorhombic V_2_O_5_ (ICDD PDF#41-1426) (see Figure 2). In the pure vanadia specimen, an additional phase of V_3_O_7_ (ICDD PDF#71-0454) is also present in minor quantities. The Zr-doped specimen also showed a fraction of V_3_O_7_ and traces of ZrV_2_O_7_ (ICDD PDF#87-0562). The Ce-doped film also presents a small fraction of V_12_O_26_ (ICDD PDF#72-1278) and traces of CeVO_4_ (ICDD PDF#72-0282). A tilting of the theta angle in the range 0.25–1.5° does not affect the relative intensities of peaks belonging to different phases, thus suggesting that different oxides are uniformly mixed together on the surface rather than ordered on different layers. The three patterns were Rietveld refined using the major phases present in the specimens (see also Section 3.1.3) and profile shape analysis was used to determine the average crystallite size. Monoclinic phase fractions were refined to be 54, 79, and 66 wt.%, respectively, for V, V-Zr2%, and V-Ce2%. In the same order, the average crystallite size values of monoclinic VO_2_ were refined to be respectively 50, 44, and 55 nm; while for V_2_O_5_ they were 50, 42, and 35 nm, respectively. In all investigated films, we never obtained a pure vanadium dioxide phase, which suggests some phase separation occurred during synthesis. Preparing procedure issues such as lower atmosphere stability of the precursors and thermal processing of the films can be ruled out for causing phase separation discrepancies. Additionally, the thermochromic transition takes place independently of the shape of the vanadia sample (film vs. powder).

The XRR results reveal a low grade of ordering in the direction perpendicular to the plane, thus dismissing the existence of a specific thickness of the films. The range of thickness was found comparable between different specimens. Additionally, differences in roughness could not be observed (Figure 2).

#### 3.1.3. What Can We See with a Temperature Increase?

**Figure 3:**
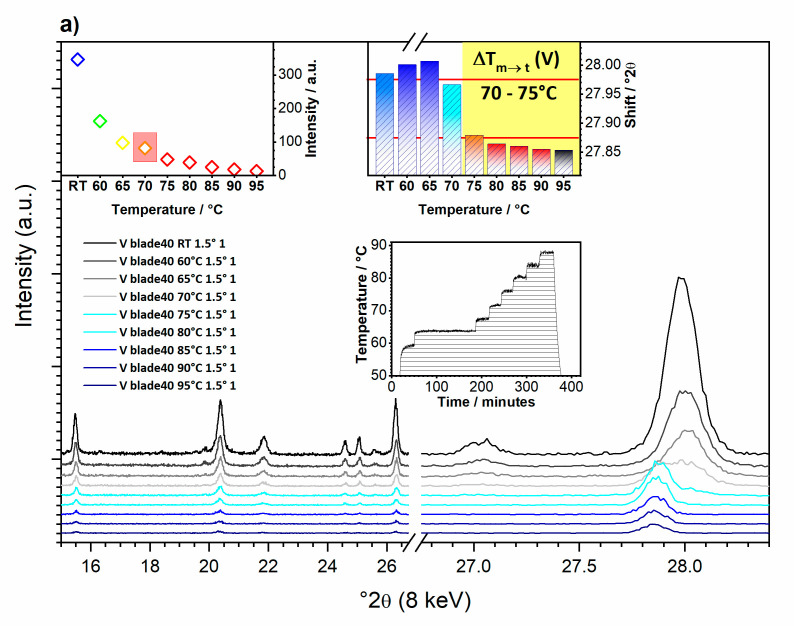

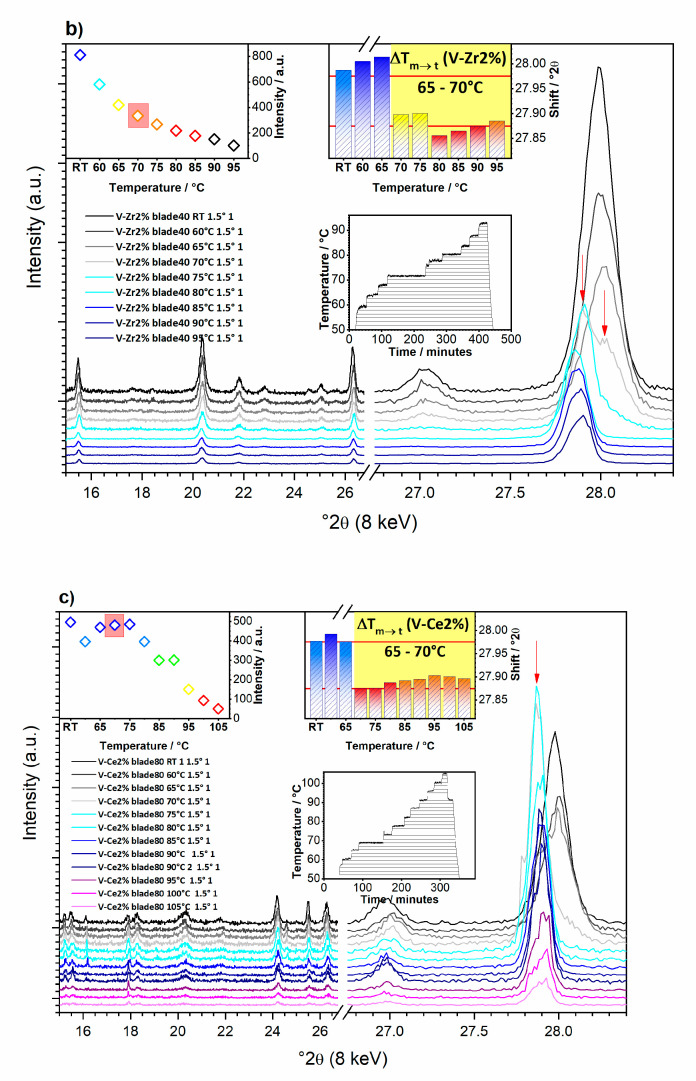
In-operando GIXRD results at 1.5° *θ*: (**a**) vanadium oxide (V), (**b**) zirconium-doped vanadium oxide (V-Zr2%), and (**c**) cerium-doped vanadium oxide (V-Ce2%). Insets: relative intensity of 011 VO_2_(M) + 110 VO_2_(T) peaks (upper left); relative shift; and thermal regime (middle).

The variation of temperature was sufficient to observe the thermochromic transition using GIXRD (see Figure 3a–c). Upon heating, the chemical composition (VO_2_) remained unchanged.

The peak at ~28° 2*θ* ((011) reflection in VO_2_(M)) better marks this transition (Figure 3a–c Inset), i.e., the unit cell parameters change due to doping and thermal expansion. This feature is related to the VO_2_ monoclinic-to-tetragonal phase change.

In pure VO_2_ this feature displays a linear trend of thermal expansion for both the monoclinic and tetragonal phases (i.e., see peak shift in Figure 3a–c). However, the tetragonal phases of V-Zr2% and V-Ce2% show an opposite trend above 80 °C, suggesting a negative thermal expansion. In the case of V-Ce2% film, the shift change returns positive above 95 °C. The overall intensity change of the diffraction is only an effect of the alignment caused by the increase of the sample stage height due to its thermal expansion during heating.

**Figure 4:**
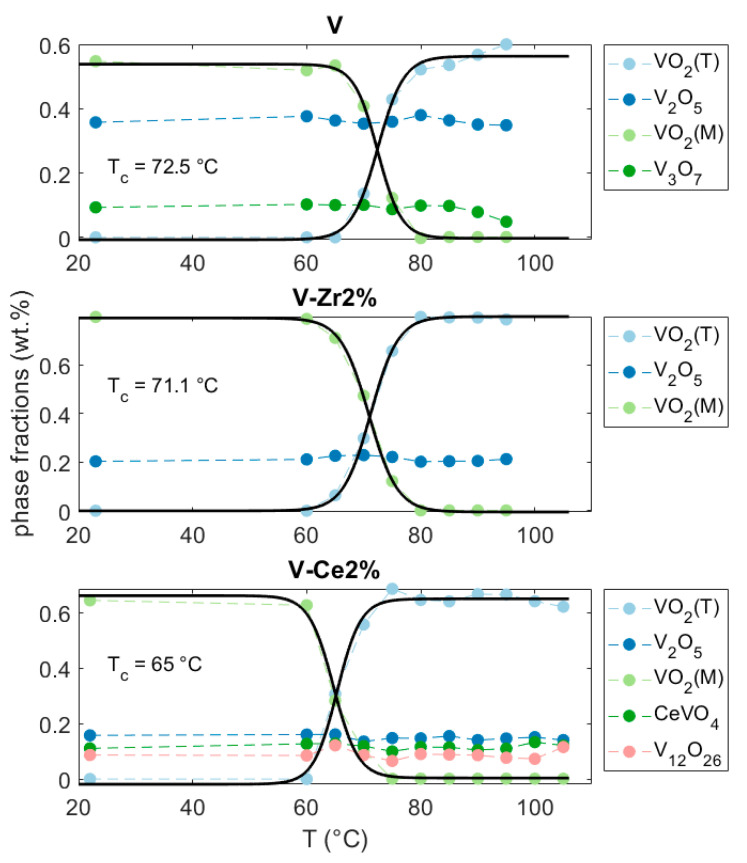
Rietveld-refined phase fractions for V, V-Zr2%, and V-Ce2% films. Solid lines represent the fitting curves as described in the text; fitted transition temperatures are reported as *T_c_*.

Generally, the fact that the phase transition occurs in the temperature range 70–75 (at 73 °C) for the pure sample, while it occurs at lower temperatures for both the Zr-doped and Ce-doped vanadia films (in the temperature range 65–70 °C), clearly shows the success of the doping for lowering the thermochromic transition (Figure 3a–c and Figure 4).

For all samples, diffraction patterns were sequentially Rietveld refined in order to follow the phase transition and the results are displayed in Figure 4. On behalf of the Rietveld refinement it is possible to follow how the fractions of the constituent crystalline phases change as a function of temperature. The fractions for V_2_O_5_, V_3_O_7_, CeVO_4_, V_12_O_26_ can be considered to be constant at all temperatures. On the other hand, the refined fractions of VO_2_(M) and VO_2_(T) were fitted to a logistic function in order to get the best estimate of the transition temperatures. The fitted transition temperatures were 73, 71, and 65 °C for V, V-Zr2%, and V-Ce2%, respectively (Figure 4). The Rietveld refinement revealed an increase in the domains average size for all films from monoclinic to tetragonal. Refined values for the tetragonal phases are determined at 80, 70, and 86 nm for V, V-Zr2%, and V-Ce2%, respectively.

**Figure 5:**
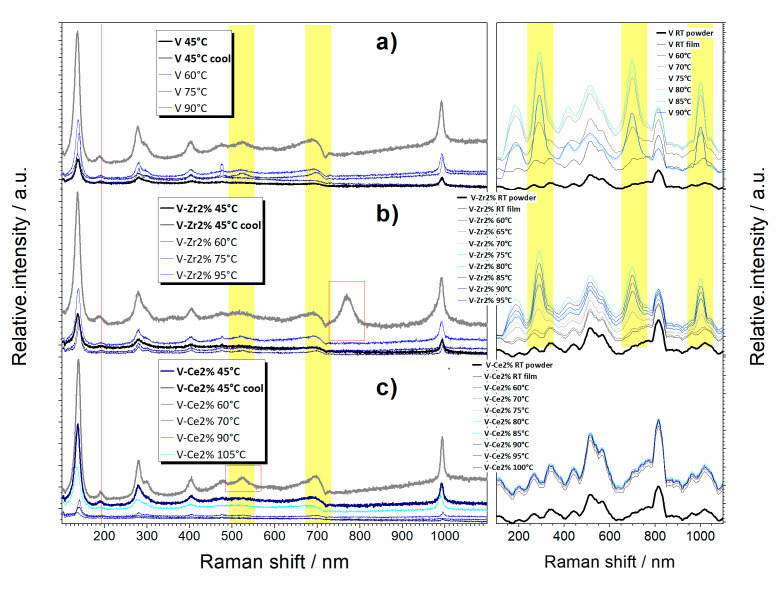
Cycling of micro Raman spectroscopy for (**a**) V, (**b**) V-Zr2%, and (**c**) V-Ce2%. Insets: cycling of in-operando Raman spectroscopy for pure, zirconium doped, and cerium-doped vanadium oxide sample.

The micro-Raman signal was intense enough to confirm the phase transition occurrence in the selected temperature range. Optical microscopy also confirms that the films’ textural properties conform well to the thermochromic functionality. More in details, even at low temperature pure, Zr- and Ce-doped films show the presence of monoclinic vanadia with a major fraction of V_2_O_5_. At high temperature, there is only a slight change in the distribution of vanadia bands for all samples. Sample V shows peaks at 145, 192, 283, 405, 483, 701, and 992 cm^−1^, which is a typical Raman spectrum of V_2_O_5_ film [24] (Figure 5). The majority of the peaks are also typical for the VO_2_ phase, so the stronger V_2_O_5_ peak may considerably hide the presence of VO_2_. The peak at 283 cm^−1^ is a two-peak superposition (283 and 303 cm^−1^), which may be the consequence of V_3_O_7_ traces. For temperatures in the range 60 to 90 °C, peaks at 523 cm^−1^ and 698–700 cm^−1^ appear, which indicate continuation of the (undesired) oxidation of VO_2_ to V_2_O_5_. Sample V-Zr2% also shows a V_2_O_5_ dominant Raman spectrum. The peak at 701 cm^−1^ shifted slightly to 696 cm^−1^ and the 188 cm^−1^ peak to 192 cm^−1^ upon heating due to Zr doping. A band appeared at 771 cm^−1^ after cooling, which was attributed to thermally induced strengthening of vanadia interface mode [25]. Sample V-Ce2% also shows a typical V_2_O_5_ dominant Raman spectrum. A band appeared at 523 cm^−1^ for heating to temperatures from 60 °C to 90 °C, which was attributed to continuation of VO_2_ to V_2_O_5_ oxidation.

#### 3.1.4. What Can We Conclude from In-Operando Measurements?

The multichannel in-operando experiment revealed the details of the thermochromic transition. First, we can conclude that the thermochromic performance of the films is not perfect. Diffraction and spectroscopic results pointed out difficulties to prepare a film with a perfect level of phase homogeneity. However, the extent of the thermochromic transition was retained. Additionally, the quality of the samples, a comparison of the doping influence, and geometric differences could be evaluated. Spectroscopic results from the portable unit, enhanced by a full-scale device, allowed a resolution suitable for low-thicknesses thin-films specimens. Doping of the VO_2_ lattice by Zr and Ce was confirmed by the successful lowering of the transition temperature by more than 7 °C. Among the doped samples, cerium-doped vanadium oxide showed lower susceptibility to phase separation, i.e., better tape casting and thermal treatment stability. This method of investigation is unquestionably fast, pragmatic, and convenient.

### 3.2. Characterisation of Bulk Powders

#### 3.2.1. Synthesis and Thermal Evolution

To confirm, evaluate, and compare the in-operando results on vanadia films, a full course of characterisation was performed on vanadia powders. For this purpose, pure and Zr-doped samples were used in a broader range of doping compositions.

**Figure 6:**
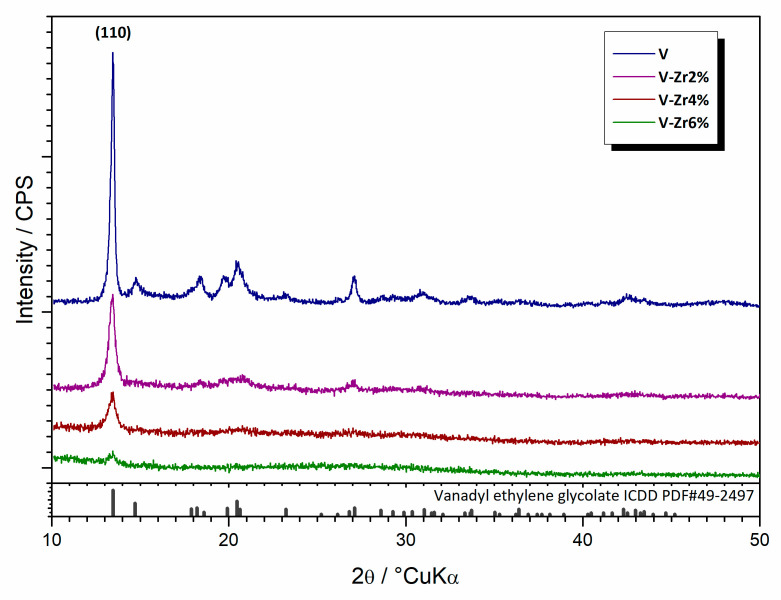
X-ray diffraction patterns of the pure V to V-Zr6% precursors samples.

The phase composition of the prepared precursor samples was determined by XRPD analysis (Figure 6). The initial step of synthesis successfully produced vanadyl glycolate, VO(OCH_2_CH_2_O), i.e., ICDD PDF#49-2497. Diffraction peaks of any other phases beside glycolate were not observed in all diffraction patterns. Bragg intensities decrease with the increase of zirconium to the point where just the (110) diffraction peak could be observed for the VO_2_-Zr6% sample, suggesting a reduction in the glycolate fraction upon doping. At the same time, the background increases, pointing to an increment of the amorphous phase.

**Figure 7:**
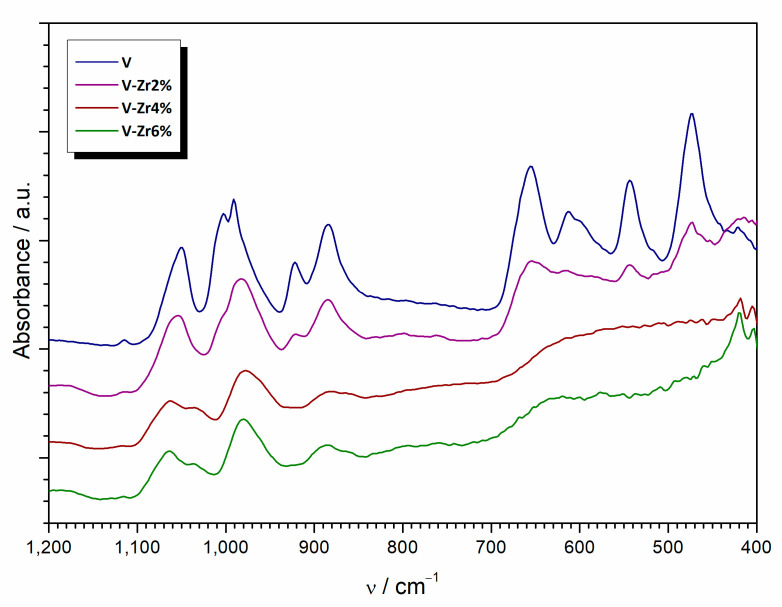
FTIR spectra of pure V to V-Zr6% precursors samples.

The FTIR spectrum of pure VO_2_ is in full agreement with the literature data for vanadyl glycolate (Figure 7). The band with a maximum at 992 cm^−1^ is related to the V=O stretching vibration in VO(OCH_2_CH_2_O) [26]. Stretching and bending vibrations of C–O occur at 1060 and 1011 cm^−1^, while C–C twisting vibrations appear at 925 and 887 cm^−1^ [27]. Peaks centred at 656 and 612 cm^−1^ potentially originate from the V–O bond [26]. With the increase of zirconium content, bands slightly shift toward greater wavenumbers and diminish in intensity. Simultaneously, new bands appear at 630 and 430 cm^−1^, attributed to the vibration of Zr–OH and Zr–O–Zr bonds, respectively [28].

From the results of XRPD and FTIR analyses, it is possible to conclude that vanadyl glycolate has been formed in each sample. However, with greater amount of zirconium butoxide added during the synthesis, greater quantity of amorphous gel has been formed. Consequently, the increase of zirconium proportion in samples decreased the overall vanadium glycol content.

**Figure 8:**
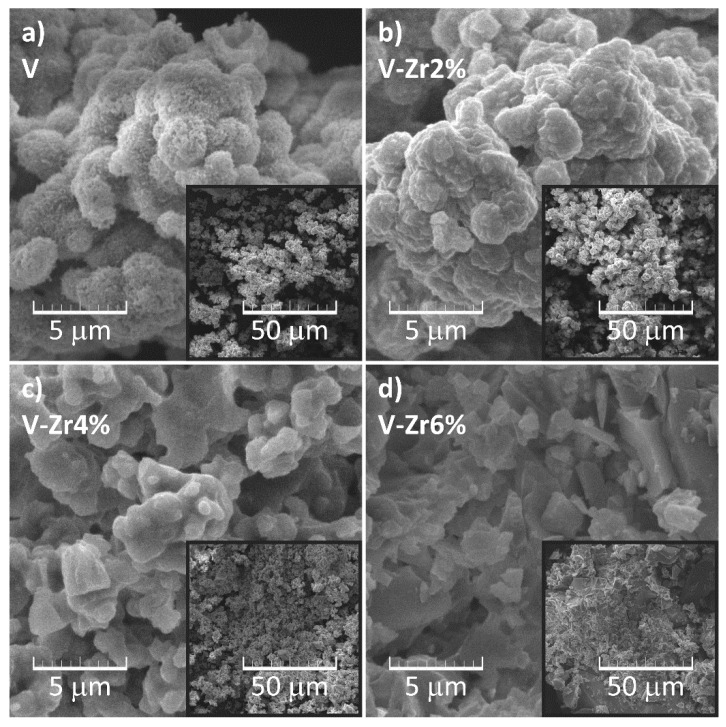
SEM micrographs of (**a**) the pure V, (**b**) V-Zr2%, (**c**) V-Zr4%, and (**d**) V-Zr6% precursor samples.

Figure 8a–d shows SEM micrographs of prepared vanadyl glycolate samples. Sample V (Figure 8a and Inset) consists of apparently spongy particles with a size up to 5 μm, which agglomerate to form secondary structures. Based on X-ray diffraction analysis, these particles can be identified as vanadyl glycolate. Krasilnikov et al. [29] reported on rod-like morphology of vanadyl glycolate, which in the present case is not observed (Figure 8a). Cao et al. [30] report a similar microstructure and explain the aggregation of vanadyl glycolate long chain structures with the urge of the system to reduce the total energy. In sample V-Zr2% (Figure 8b and Inset) the particles are more compact. Based on XRPD analysis and FTIR spectroscopy, it can be concluded that these particles comprise of vanadium glycolate and amorphous gel. From the micrograph of the sample V-Zr4% (Figure 8c and Inset) it is apparent that the amorphous gel share in this sample is larger than that in the V-Zr2% sample because the particles at a larger magnification (Figure 8c) seem smoother. This observation is fully consistent with the XRPD results and infrared spectroscopy, suggesting a lower proportion of vanadyl glycolate and higher gel content in this sample. The micrographs of the V-Zr6% (Figure 8d and Inset) are typical for xerogels, where even the cracks that appeared due to gel drying can be noted. This observation is also in full compliance with the results of XRPD analysis and FTIR spectroscopy indicating a negligible portion of glycolate, i.e., a dominant gel content in this sample.

**Figure 9:**
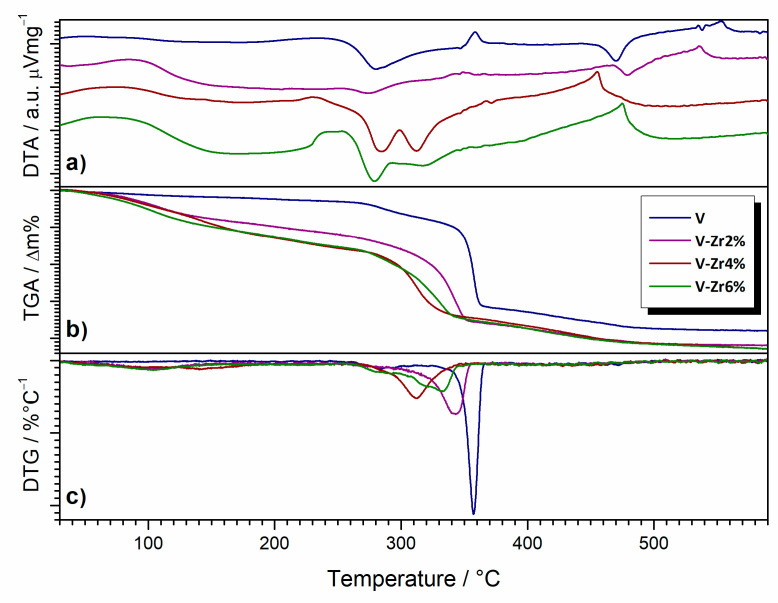
(**a**) DTA, (**b**) TGA, and (**c**) DTGA curves of the pure V to V-Zr6% samples.

Figure 9 shows differential thermal analysis (DTA), thermogravimetric analysis (TGA) and differential thermogravimetric analysis (DTGA) curves of the prepared samples. On the DTA curve of sample V one could observe a very weak endothermic process between 50 and 150 °C, followed by an exothermic effect ranging between 230 and 330 °C. In the range of 330–370 °C, a weaker endothermic process takes place, followed by an exothermic peak in the region of 440–500 °C, and, finally, an endothermic event between 530 and 570 °C. On the TGA curve, there is a slight mass loss from room temperature to 150 °C, followed by a significant mass loss in several stages ending at ~500 °C. From the DTGA curve, one can see that in the temperature range 150–240 °C there is a continuous mass loss, followed by a weaker mass loss in the range 240–320 °C and one more intense mass loss in the range 320–370 °C. Finally, there is a continuous loss of mass in the range of 370–500 °C. V and V-Zr2% DTA curves are very similar. At DTA curves in the temperature range of 240–320 °C for samples V and V-Zr2% a single exothermic effect could be observed while for V-Zr4% and V-Zr6% two exothermic effects were observed. However, for the V sample a single endothermic event takes place in the range of 350–370 °C and shifts to lower temperatures and decreases in intensity for doping increase. In samples with higher doping contents, this effect might be cancelled by the appearing of the aforementioned second exothermic peak. However, the stronger endothermic effect appearing in the range of 530–570 °C for samples V and V-Zr2% seems to be shifted to 400–500 °C for V-Zr4% and V-Zr6%. In the 400–500 °C temperature range, a single exothermic peak appears only for samples V and V-Zr2%. The exothermic effect noted in this temperature range for samples V and V-Zr2% was possibly concealed for samples V-Zr4% and V-Zr6% by the intense aforementioned endothermic effect. TGA curves of samples V-Zr2%, V-Zr4% and V-Zr6% are similar to the one of the V sample but all processes are shifted to lower temperatures and the total mass loss is greater. The majority of these differences arise from room temperature to 300 °C.

**Figure 10:**
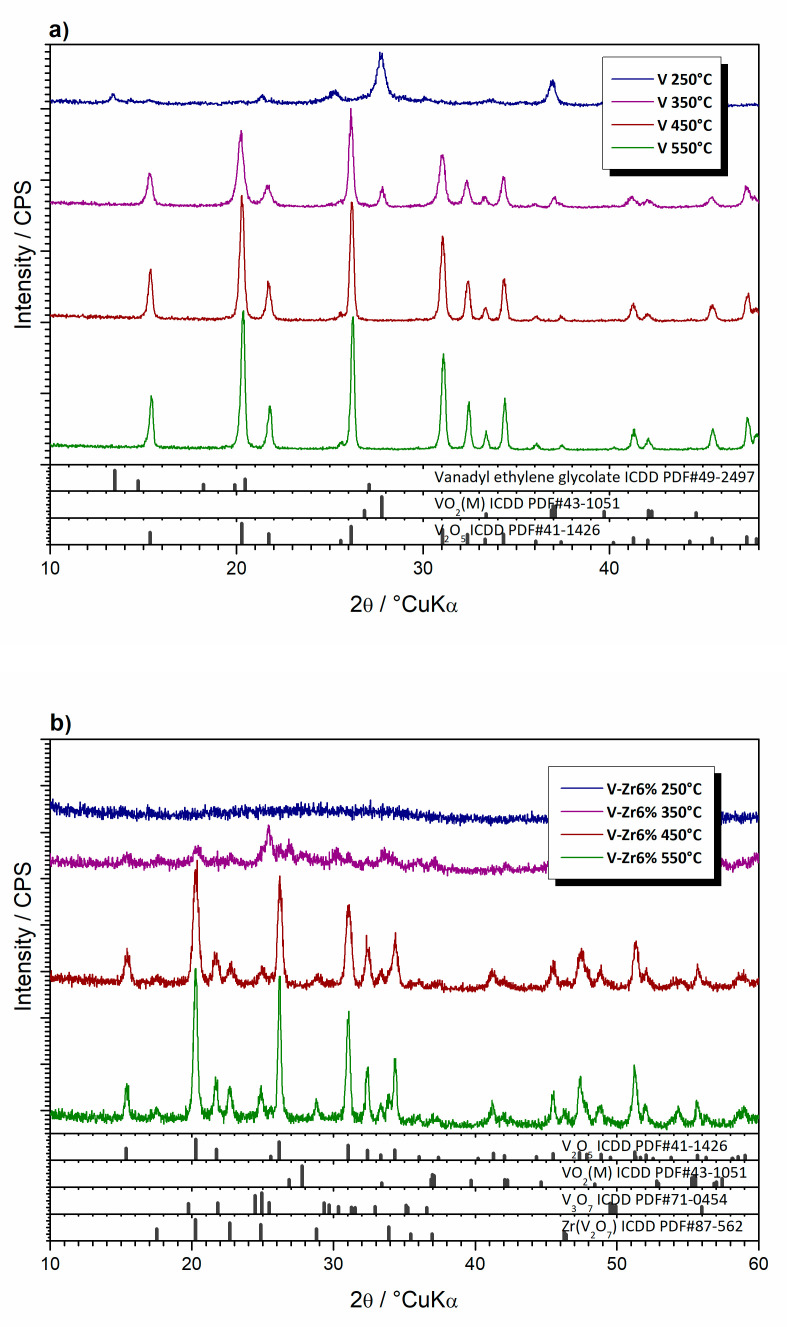
X-ray diffraction patterns of samples (**a**) pure V and (**b**) V-Zr6%, the thermal treatment of which was interrupted at 250, 350, 450, and 550 °C.

To gain a better insight into the thermal evolution of the prepared vanadium glycolate and ascribe the processes noticed by thermal analysis, the prepared samples were heated to different temperatures in a laboratory furnace under static air atmosphere and then subjected to XRPD analysis (Figure 10). Notably, the conditions in the furnace are not exactly equal to those in the DTA/TGA apparatus in terms of atmosphere and dynamics, as well as temperature accuracy, which is inferior in the case of the oven compared to the DTA/TGA apparatus. From the diffraction patterns of sample V (Figure 10a), it is apparent that after annealing to 250 °C, a complete decomposition of VO(OCH_2_CH_2_O) accompanied by formation of VO_2_ occurred. Diffraction peaks are typical of VO_2_(M), ICDD PDF#43-1051. This phase is stable below 68 °C, thus in fact VO_2_(T) crystallised first and then was transformed to VO_2_(M) as the effect of cooling the sample before the ex-situ XRPD analysis. Crystallite sizes, calculated using the Scherrer’s equation were found to be 40 and 126 nm, respectively for V samples treated at 250 and 350 °C. Even though VO_2_(M) diminishes quantitatively, the crystallite size increases strongly. Upon heating to 350 °C, a new V_2_O_5_ phase, ICDD PDF#41-1426, appears. V_2_O_5_ also exhibits a semiconductor to metal transition in a temperature range from 250–280 °C [31]. Similarly, the observed semiconductor phase was formed by transformation in the course of cooling from the metallic phase that first crystallised. Up to 450 °C, VO_2_ oxidation to V_2_O_5_ is complete. The XRPD pattern of the sample heated up to 550 °C does not show the appearance of any new phase, and this sample, as well as the one interrupted at 450 °C, consists only of V_2_O_5_. The narrowing of V_2_O_5_ diffraction peaks with the increase in the annealing temperature at which thermal treatment of the sample V was interrupted points out to V_2_O_5_ crystallites growth. After heating to 250 °C, the sample V-Zr6% is completely amorphous (Figure 10b), while annealing at 350 °C, produces Zr(V_2_O_7_) (ICDD PDF#87-0562), V_3_O_7_ (ICDD PDF#71-0454), and V_2_O_5_ in minute amounts. In patterns of samples annealed at 450 °C or 550 °C, these diffraction peaks become narrower pointing out to crystallites growth. By further heating both samples were melted, as expected, since V_2_O_5_ melts at 690 °C [27].

**Figure 11:**
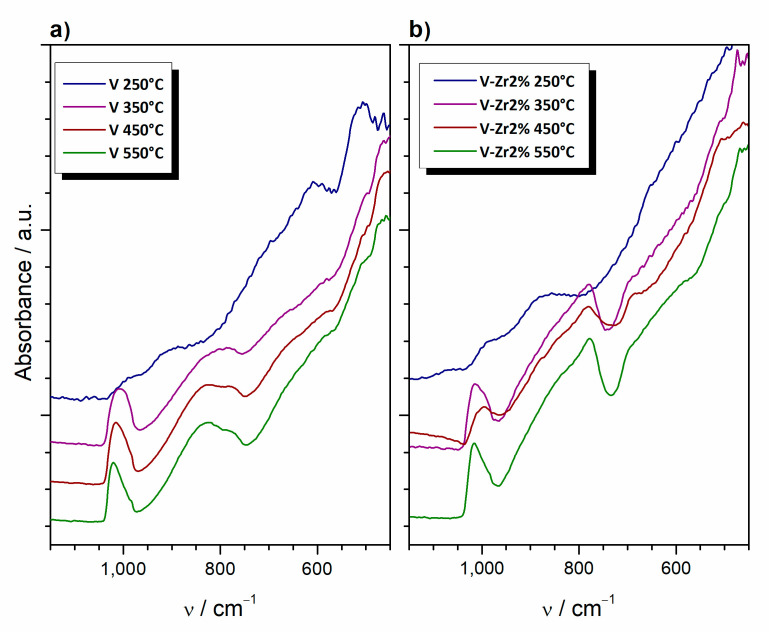
FTIR spectra of the samples (**a**) pure V and (**b**) V-Zr6%, whose thermal treatment was interrupted at temperatures of 250, 350, 450 and 550 °C.

To gain additional information on the thermal evolution of vanadium glycolate, FTIR analysis was carried out on V and V-Zr6% samples, with heating interrupted at different temperatures. Figure 11 shows only a segment of the spectrum between 1200 and 400 cm^−1^ where relevant absorption bands appear. In this area, the spectrum of pure VO_2_ processed at 250 °C displays two bands at 605 and 505 cm^−1^, both attributed to octahedral V–O–V bending in VO_2_ [32,33]. Undoped samples thermally treated at higher temperatures are characterised by bands at 1015 and 825 cm^−1^. According to Slurca and Orel [34] and Farahmandjou and Abeiyan [35], those bands are typical for V_2_O_5_, with bands appearing between 950 and 1020 cm^−1^ corresponding to the V–O (vanadyl) stretching modes and bands between 700 and 900 cm^−1^ to the bridging V–O–V stretching. Additionally, Farahmandjou and Abeiyan ascribe the band at 730 cm^−1^ to the V–O–V asymmetric stretching. FTIR spectra of the doped sample show a band at 785 cm^−1^, which corresponds to vibrations of the Zr–O bond [36].

Based on XRPD analysis, DTA and TGA curves can be explained as follows. The weak endothermic process between 50 and 150 °C, accompanied by a certain mass loss, is the result of the adsorbed moisture and evaporation of residual organic phases. Significantly higher mass loss in this temperature range for the samples prepared using zirconium butoxide (in which the gel was formed) is obviously the consequence of the gel decomposition. The exothermic effect in the range 230–320 °C, followed by a certain mass loss, is the result of glycolate decomposition and crystallisation to vanadium oxides, in particular VO_2_ and V_2_O_5_, as well as Zr(V_2_O_7_) in samples containing zirconium. Cao et al. [30] also report on an exothermic DSC peak at 253 °C, and corresponding sharp mass loss in TGA curve, which attribute to vanadyl glycolate decomposition to VO_2_. The weak endothermic event on the DTA curve of sample V in the range 330–370 °C, which appears along with a great mass loss, is obviously a consequence of the rapid decomposition and release of the remaining organic phase from the sample. In all other samples, this process occurs in parallel with the crystallisation of vanadium oxides, so the endothermic process is probably superimposed to the exothermic process of crystallisation. According to Zhang et al. [37] oxidation of VO_2_ to V_2_O_5_ is an exothermal process and the thermal range of its occurrence greatly depends on the VO_2_ polymorph. Based on this data and XRPD measurements, the exothermal effect at 450 °C is attributed to the oxidation of VO_2_ to V_2_O_5_. This effect is not observed in DTA curves for V-Zr4% and V-Zr6% since VO_2_ forms only in limited quantities in these samples. The oxidation process should be accompanied by a mass gain; however, it was not detected owing to the overlapping of this process with the release of the sample disintegration products. Other authors also report that this process is not clearly visible due to overlapping with other effects [29]. No obvious reasons exist for the occurrence of the last endothermic peak and corresponding mass loss appearing between 450 and 550 °C. The appearance of Zr(V_2_O_7_) diffraction peaks in the diffraction patterns of V-Zr6% indicate that in the described processes of synthesis and thermal treatment zirconium enters the crystalline lattice of VO_2_ in a limited amount.

#### 3.2.2. Thermally Treated Powders

Based on the results reported so far, the best temperature to synthesize VO_2_ would be 250 °C. However, the full elimination of organic residuals requires annealing up to 400 °C. To avoid further oxidation of VO_2_ to V_2_O_5_ and other vanadium oxides, the samples were inserted in a furnace previously heated to 400 °C and held for a short time (5–10 min).

**Figure 12:**
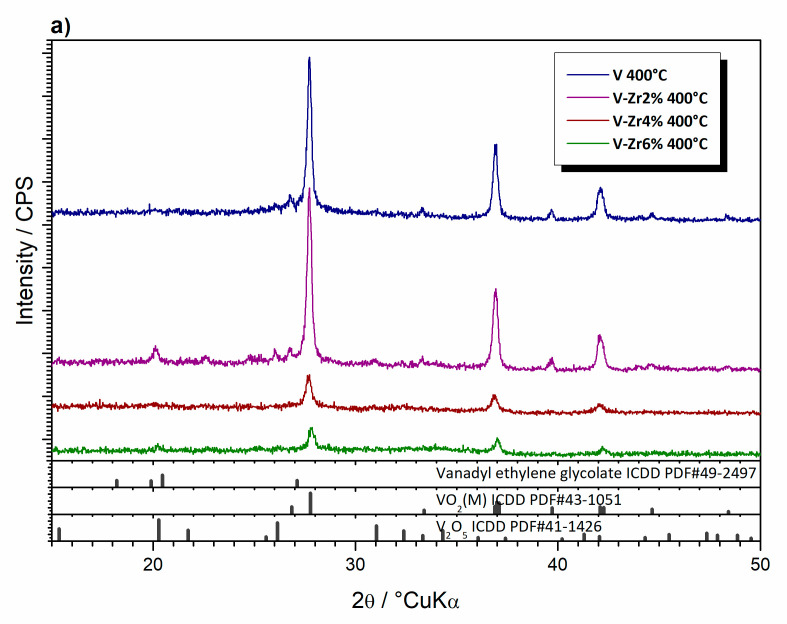

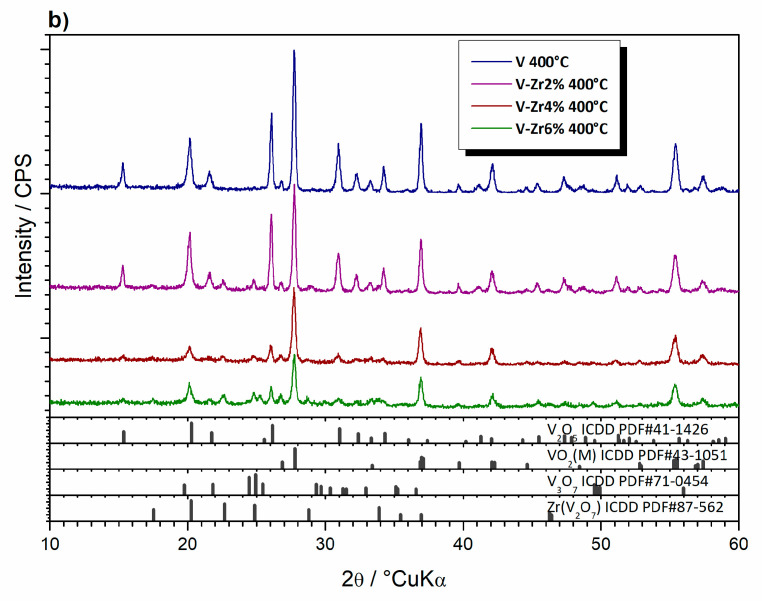
X-ray diffraction patterns of the pure V to V-Zr6% powder samples thermally treated at 400 °C for (**a**) 5, (**b**) 10 min.

After thermal treatment at 400 °C for 5 min, only diffraction peaks of VO_2_(M) were observed in sample V (Figure 12a). However, in the diffraction pattern of V-Zr2%, alongside with VO_2_(M), very weak features of V_2_O_5_ and V_3_O_7_ could be observed. In the case of V-Zr4% and V-Zr6%, VO_2_(M) is dominant. Further annealing up to 10 min leads to crystallisation of several phases in all samples (Figure 12b). VO_2_(M) and V_2_O_5_ are present in sample V, and V_3_O_7_ and ZrV_2_O_7_ also appear in all other samples. Crystallite sizes, calculated using Scherrer’s equation, for VO_2_(M) phase in samples V to V-Zr6% thermally treated at 400 °C for 5 min are 140, 122, 84, and 77 nm, respectively. For the same samples treated for a further 10 min, the sizes are 146, 132, 110, and 103 nm. From these results it appears that doping hinders the growth of the crystallites whereas a longer thermal treatment promotes their growth also leading to formation of other oxides (Table 2).

**Figure 13:**
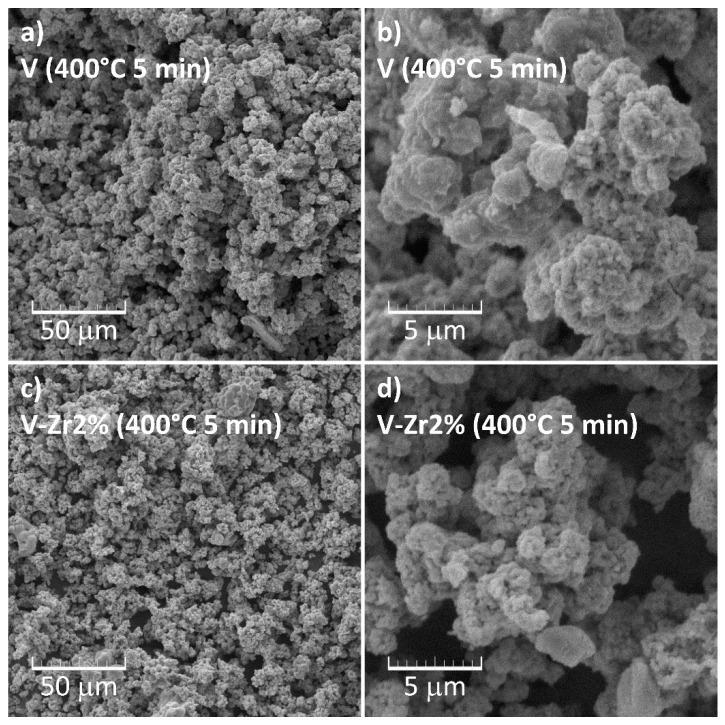
SEM micrographs of (**a**) pure V at lower magnification, (**b**) pure V at higher magnification, as well as (**c**) V-Zr2% at lower magnification, (**d**) V-Zr2% at higher magnification, as obtained after treatment at 400 °C for 5 min.

Micrographs of the samples thermally treated at 400 °C for only 5 min (Figure 13) clearly confirm that the initial morphology of vanadyl glycolate is partially preserved, whereas traces of gel morphology are not apparent. The microstructure of V and V-Zr2% is quite similar. The evaluation of formed particles in terms of dimension is difficult due to extensive agglomeration.

**Figure 14:**
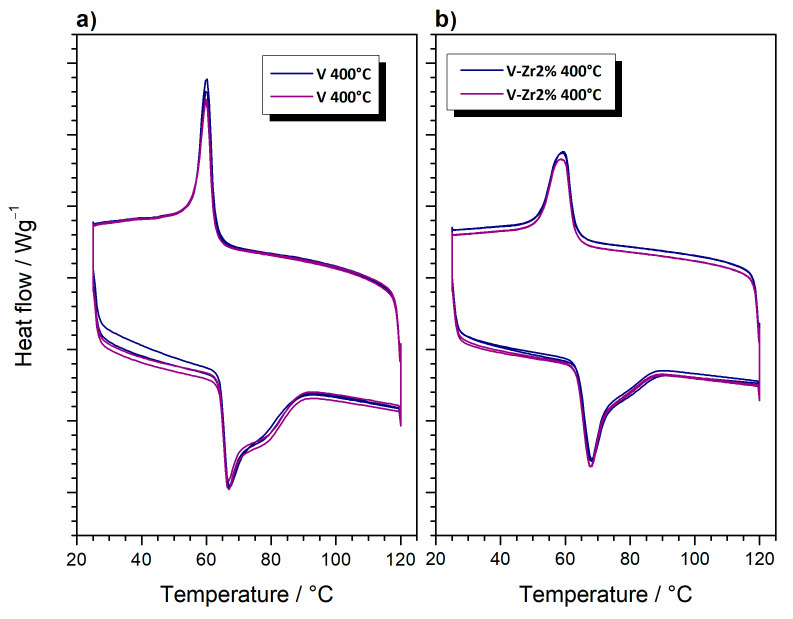
DSC curves of VO_2_ prepared from samples (**a**) pure V and (**b**) V-Zr2%, by thermal treatment at 400 °C for 5 min; bottom curves—heating, upper curves—cooling.

Figure 14 shows DSC curves of VO_2_ samples obtained through four heating and cooling cycles. The reversible polymorphic transition process from semiconducting VO_2_(M) to metallic VO_2_(T) and vice versa [32] are clearly observable. The notable hysteresis is due to the latent heat release or adsorption during the first order phase transition [38]. The transition temperature, taken as the maximum of the endothermic process accompanying the transition from the monoclinic to the tetragonal, is 67 and 68 °C for V and V-Zr2%, respectively. These values are very close to that reported in the literature for this transition, which is 68 °C [39]. Upon cooling, exothermic processes are found at 60 and 59 °C for V and V-Zr2% respectively, also close to the literature values. The expected reduction in the transition temperature due to the addition of zirconium [40] is limited, which points to the fact that incorporation of zirconium in the crystal lattice of VO_2_ is not substantial. A shoulder in the high-temperature side of the endothermic peak appears for both samples during heating and is a consequence of polydispersity [41]. Furthermore, a reduction of the DSC curve areas of both processes for sample V-Zr2% in comparison with sample V, can be observed. Generally, the decrease of phase transition DSC effect magnitude with increasing dopant concentration is a known phenomenon occurring in VO_2_ doped with heavy atoms [42]. Such behaviour is usually interpreted as being a consequence of the effective doping (entrance of a dopant in a crystal lattice of VO_2_). However, in the present case, the reduction of the transition peaks magnitude is most likely caused by the presence of V_2_O_5_ and V_3_O_7_ in V-Zr2%. The appearance of those vanadium oxides in sample V-Zr2% has been established by XRPD analysis (see Figure 12). The decrease of VO_2_ in sample V-Zr2% thus causes the small drop in the DSC peaks magnitude. DSC also allows to estimate the stability of the prepared material since it provides insight about the changes in temperature and enthalpy of the phase change process after several heating and cooling cycles. From Figure 14, slight changes in the maximum temperature and the peak area, proportional to the enthalpy, can be observed, only occurring after the first cycle and no further. This is just a consequence of the different thermal histories of the samples before and after the first heating cycle. Without differences in thermal history, no differences in peak temperature or area exist. Four stable heating and cooling cycles can serve as a reliable indication to prove that the prepared VO_2_ is thermally stable.

## 4. Conclusions

Through the reaction of ammonium metavanadate and ethylene glycol, intermediate vanadium glycolate was prepared. The proportion of glycolate in the samples rapidly decreases with doping, so the sample in which 6% of vanadium is replaced by zirconium is almost entirely amorphous.

Thermal and structural analyses of thermally treated samples show that pure glycolate decomposes to VO_2_, which is further transformed into V_2_O_5_ in air. For Zr-doped samples, Zr(V_2_O_7_) and V_2_O_5_ crystallize first, followed by V_3_O_7_.

Uncontrolled thermolysis of glycolate sample results in a mixture of VO*_x_* crystalline phases, but thermal processing of pure vanadia sample at 400 °C for 5 min allows pure VO_2_ to be obtained.

In powdered samples, only a minor deviation of the thermochromic transition temperature (temperature shift of DSC maximum) was observed, compared to the literature data, resulting from the reversible polymorphic transition of VO_2_(M) to VO_2_(T) and vice versa, ultimately pointing out to limited doping of the metal ions in the crystal lattice of VO_2_ (minor transition temperature decrease and phase separation).

Thin-film deposition onto glass proved to be a successful process for the majority of the samples. The doping type and quantity affect the crystallite and particle sizes as well as the specific surface.

Using a multichannel in-operando setup we revealed the structural details of the thermochromic transition. Diffraction and spectroscopic results pointed out that the prepared films had a considerable level of chemical homogeneity, whereas spectroscopic results enhanced by a full-scale device allowed resolution suitable for thin-films with low-thicknesses. The fact that the transition temperature was lowered on average by 5 °C suggests that metal ions doping in VO_2_ was successful from the structural point of view. Among doped samples, Ce-VO_2_ showed only minute phase separation, better tape casting and thermal treatment stability and consequently more favourable structure modification. Primarily on behalf of structural influence, the derived thin-films (especially Ce-VO_2_) can thereof be considered as candidates for materials with better thermochromic behaviour. The method of synthesis is unquestionably fast, pragmatic, and convenient. Ultimately, the exploited diffraction/spectroscopic thermal in-operando setup enables comprehensive self-standing characterisation on nanoscale materials, including thermochromic films.

## Figures and Tables

**Table 1:** Investigated samples. Thermochromic smart window samples denomination; pure vanadia, Zr-doped vanadia, and Ce-doped vanadia.

Sample	Composition	Type	Thermal Treatment (°C, min)
V	VO_2_	powder	150, 60
V-Zr2%	V_0.98_Zr_0.02_O_2_	powder	150, 60
V-Zr4%	V_0.96_Zr_0.04_O_2_	powder	150, 60
V-Zr6%	V_0.94_Zr_0.06_O_2_	powder	150, 60
V	VO_2_	film	100, 360
V-Zr2%	V_0.98_Zr_0.02_O_2_	film	100, 360
V-Ce2%	V_0.98_Ce_0.02_O_2_	film	100, 360

**Table 2:** Properties of the samples. Thermochromic smart window parameters of all powder samples—pure vanadia and Zr-doped vanadia (2, 4, 6%) (annealed at 400 °C for 5 min)—and film samples—pure vanadia, Zr-doped vanadia (2%) and Ce-doped vanadia (2%) (annealed at 100 °C for 360 min). The amount of “•” symbolises an arbitrary unit representing purely empiric interpretation of combination of all investigated factors and parameters that are prerequisite for achieving thermochromic quality.

Sample	Type	Crystallites (nm)	Specific Surface Area (Arbitrary Units)	Film Thickness (μm)	Thermochromic Quality (Arbitrary Units)
V	powder	140	moderate	-	•••••
V-Zr2%	powder	122	moderate	-	••••
V-Zr4%	powder	84	high	-	••
V-Zr6%	powder	77	high	-	•
V	film	50	-	24	•••
V-Zr2%	film	44	-	24	••••
V-Ce2%	film	55	-	24	•••••
